# The Association between Health and Culture: The Perspective of Older Adult Hospital In-Patients in Israel

**DOI:** 10.3390/ijerph18126496

**Published:** 2021-06-16

**Authors:** Ahuva Even-Zohar, Varda Shtanger, Anat Israeli, Emma Averbuch, Gad Segal, Haim Mayan, Shmuel Steinlauf, Alex Galper, Eyal Zimlichman

**Affiliations:** 1 Faculty of Social Sciences, School of Social Work, Ariel University, Ariel 40700, Israel; 2 Quality Assurance Department Patient Report Outcome Measures, Sheba Medical Center, Tel Ha Shomer 52620, Israel; Varda.stenger@sheba.health.gov.il; 3 Internal Nursing Wing, Sheba Medical Center, Tel Ha Shomer 52620, Israel; Anat.Israeli@sheba.health.gov.il; 4 Reduction of Health Inequalities Unit in the Administration for Strategic and Economic Planning at the Israeli Ministry of Health, Jerusalem 9101002, Israel; Emma.Averbuch@MOH.GOV.IL; 5 Internal Medicine, Sheba Medical Center, Tel Ha Shomer 52620, Israel; Gad.Segal@sheba.health.gov.il (G.S.); Haim.Maayan@sheba.health.gov.il (H.M.); Shmuel.Steinlauf@sheba.health.gov.il (S.S.); 6 Quality Assurance Department Patient Report Outcome Measures, Tele-Health Project, Sheba Medical Center, Tel Ha Shomer 52620, Israel; Alex.Galper@sheba.health.gov.il; 7 Innovative and Quality Assurance Department, Sheba Medical Center, Tel Ha Shomer 52620, Israel; Eyal.Zimlichman@sheba.health.gov.il; 8 Sackler School of Medicine, Tel Aviv University, Tel Aviv 6997801, Israel

**Keywords:** health, culture, older adults, hospital in-patients, ageism

## Abstract

People from different cultures are often hospitalized while the staff treating them do not have sufficient knowledge about the attitudes and feelings of the patients regarding culture and health. To fill this gap, the aim of this study was to examine the perspective of Israeli older adult hospital in-patients regarding the association between health and culture and to understand the meaning of the participants’ experiences with regards to the medical staff’s attitude towards them. This study was carried out using qualitative methodology that followed the interpretive interactionism approach. The research participants were 493 (mean age 70.81, S.D.: 15.88) in-patients at internal care departments at a hospital in Israel who answered an open-ended question included in the questionnaire as part of a wide study held during 2017 to 2018. Two main themes were found: (1) a humane attitude of respect and the right to privacy and (2) beliefs, values, and traditional medicine that are passed down through generations. The findings highlighted the issue of the patients’ cultural heritage and ageist attitudes they ascribed to the professional staff. This study provided recommendations for training the in-patient hospital workforce on the topic of cultural competence, beginning from the stage of diagnosis through treatment and to discharge from the hospital, in order to improve the service.

## 1. Introduction

Hospitals and community-based health facilities are often centers of multicultural integration between people from different backgrounds, whether members of the nursing, paramedical, or medical staff or the patients themselves [1]. This is especially true in Israel, where there is a variety of different cultures reflected in different beliefs, philosophies, religions, origins, languages, and values [2,3]. Indeed, the residents of Israel, where this study was conducted, belong to different ethnic groups and cultures with diverse origins: Jews who immigrated to the country from both Western and Eastern countries side-by-side with minorities: Muslim Arabs, Christian Arabs, and Druze [4]. 

Multicultural studies indicate that, in cases of diverse culture, there are linguistic and communication barriers that lead to deficient communication for both patients and medical staff [5]. Patients are less likely to report symptoms. There may be poor communication due to language and communication barriers; a lack of support networks; unfamiliar values, customs, and beliefs; different cultural backgrounds; and an unfamiliarity with and lack of understanding of patients’ cultural backgrounds [6]. Consequently, sometimes there is less a chance of understanding the nature of the treatment and of receiving an explanation of the follow-up recommendations [7]. An incorrect diagnosis may be given due to communication difficulties, which increases the chance of medical errors and may delay treatment, and there is a chance that the patient will not follow instructions [8].

In addition, people are concerned about presenting for medical care and, hence, avoid treatment, with a consequent delay in providing care. Among patients from different cultures, less use of primary care and emergency room visits has been shown [8] and, on the other hand, repeated visits to the hospital due to misunderstandings, and there is a higher likelihood of violent behavior and less satisfaction with the treatment provided by healthcare providers. Attending to the cultural constructions of health opens up the discursive spaces of health communication to alternative cosmologies of health, illness, healing, and curing [9]. The problem of culture is, in fact, a problem of understanding relationality, oneself, and other people, and it is important to understand all those within the therapeutic environment (family, patients, and professionals) [10].

Cultural differences also have implications for the duration of patients’ hospital stays. For example, a study [11] conducted in the United States examined the relationship between the length of one’s hospital stay and repeated admissions among patients with a limited knowledge of English. According to the study, among those patients who did not receive an explanation in their first language when entering and leaving the hospital, the length of stay ranged from 0.7 to 4.3 days more than among those whose first language was English. In addition, these patients tended to come for more return visits [11]. In Israel as well, there are population groups whose first language is not Hebrew, and they experience difficulties with receiving healthcare due to problems with speaking Hebrew.

Cultural competence is one of the main strategies currently recognized by Western health organizations for addressing the issues related to multicultural population care [12]. Cultural competence is defined as “behaviours, characteristics, and procedures that allow an institution (or individual) to function effectively in multicultural situations” [13]. There are several stages leading to cultural competence: “cultural awareness” (self-examination of beliefs, values, prejudices, perceptions, and communication patterns); “cultural knowledge” (expanding knowledge and familiarity with cultural characteristics of minorities, groups, and sectors); “cultural sensitivity” (developing sensitivity, empathy, understanding, and awareness of others and their differences in society and the community); and “cultural implementation” (the ability to collect relevant cultural data on disease history and to perform tailored physical examinations) [13]. 

The use of this strategy is reflected in the application of cultural accessibility tactics and the development of tools and skills and their implementation in the field [13]. For instance, in the United States, which is an ethnically diverse country, high levels of change in knowledge competence and performance were reported as a result of cultural competence training in organizations [14]. In addition, physician communication behaviors may have a varying effect on the patient’s trust, depending on the patient’s race. Indeed, communication skills training programs for physicians targeting emotion handling and rapport building behaviors are promising strategies for reducing the disparities in healthcare and enhancing trust among ethnic minority patients [15].

The Israeli Ministry of Health has defined activities to minimize health inequalities as a strategic goal and is working to reduce the disparities in healthcare and health services. In the past decade, reports have been published describing a variety of cultural competence training and implementation programs for medical teams. These programs focus on minority populations and address language difficulties, racial attitudes, and improving the cultural competence of the medical team [16].

A Ministry of Health protocol from 2011 set the standards regarding access to health services in a way that can overcome cultural and linguistic barriers. This was intended, among other things, to improve the compliance with treatments and lead to an improved quality of service for patients. In order to provide culturally and language-adapted care, there is a clear need to raise personal and social awareness, as well as to effect a change in attitudes and perceptions, procedures, and regulations in the hospital setting [16]. This can be achieved by developing cultural, educational, and training programs for medical service providers, as well as by the continuous evaluation of the cultural competence training program.

The current study is part of a wide intervention study held during 2017 to 2018 at a hospital in Central Israel with the purpose of investigating the impact of an intervention program for cultural accessibility on the staff and patients, particularly with respect to communications, treatments, and explanations of diagnoses and treatments [17]. The current study highlighted the issue of the patients’ cultural heritage through answers to an open-ended question included in a cultural heritage questionnaire [6,18] exploring patients’ family, religious, and social educational heritages. In this study, we chose to focus on older people, since most of the patients who were hospitalized at the time in the departments examined were older than 65.

## 2. Methods

The research question focused on the participants’ description of the association between health and culture. Since the purpose of the study was to thoroughly understand the patients’ cultural heritage, experiences, perceptions, and attitudes, the research method chosen was the qualitative method [19]. The study followed the interpretive interactionism approach [20,21,22], with the aim of understanding the meaning of the participants’ experiences while in the hospital with regards to the medical staff’s cultural attitude towards them. 

This approach focused on the individual’s experiences and interpretations of these experiences, taking into account the interrelations with the social environment. Utilizing the perspectives of the participants and understanding their experiences can affect the discourse and interaction between the medical staff and the patients and can contribute to developing the policy of the healthcare system at large. The interpretive interactionism approach makes it possible to situate the research findings within the health policy, professional ethics, and organizational approaches to care, because this approach constitutes a linking and placement of client experiences within a social and organizational structure [22].

Aiming to document and conceptualize in-patient perspectives regarding health and culture in light of staff workshops on cultural accessibility, our research question was: How do you view the association between health and culture? As the research question suggests, our aim was to learn about the experience of in-patients. Thus, among the different approaches to qualitative research, e.g., the grounded theory, we chose the interpretative interactionism approach [19]. This approach aims to understand the participants themselves—that is, people’s lived experiences and the meanings they attach to their experiences. Therefore, it is suitable for the present study that focuses on the meanings attributed by patients as they interact with the healthcare and social systems and can help the research process clarify the experiences of the patients, especially by conducting studies on social and personal problems that have policy implications [18,19].

### 2.1. Participants

The research participants were in-patients sampled at random at three internal care departments at a hospital in Central Israel, all with a similar size and patient population. The participants were suffering from illnesses such as cancer, cardiovascular illness, gastrointestinal disorders, and metabolic illnesses. At the time (2017 to 2018), workshops on cultural and linguistic competences were being conducted for physicians and nurses in accordance with a guidance kit provided by the Ministry of Health (www.http.msr.org.il, accessed on 19 January 2021). 

The participants interviewed were sampled in a convenience sample. Inclusion criteria: all the in-patients at an internal care department one day before discharge. Exclusion criteria: demented patients and those who refused to be interviewed. There was a total of 493 participants: 271 women (55.0%) and 222 men (45%). Most were older than 65, and their mean age was 70.81 (S.D.: 15.88). Regarding their marital status, 57.4% were married, 21.5% were widows, 14.2% were divorced or separated, and 6.9% were single. Regarding ethnicity, 96.4% were Jews, 1.8% were Muslims, 1.6% were Christians, and 0.2% were Druze. Of the Jews, one-third were religious, one-third were traditional (the traditional population observes certain religious laws and a number of Jewish rituals and customs, such as celebrating the High Holy Days), and one-third were secular. Eighty percent were Hebrew speakers, 15.6% were Russian speakers, 3.0% were Arabic speakers, and 1.4% were Amharic speakers. Three hundred and fifteen questionnaires were completed by the participants (63.89%) and 178 by a companion (36.11%). Of all the questionnaires in which the open-ended question was answered, 289 patients (59%) answered this question at length.

### 2.2. Research Tool

Towards their discharge from the hospital, the participants were asked to answer an open-ended question: How do you view the association between health and culture? This open-ended question was part of a broader questionnaire on cultural heritage [6,18]. The broader questionnaire included closed-end questions that related to gender; education; country of birth; age of immigration to Israel for those who were not born in Israel; language preferences for receiving healthcare (speaking, reading, and writing); religion and religious observances; and preparing ethnic foods.

### 2.3. Ethical Aspects

This study was conducted after receiving the approval of the hospital’s Helsinki Committee, approval no: 2081-15-SMC. An appeal was made to in-patients in the departments by the research team (researchers and research assistants who were not part of the medical staff), asking them to participate in the study. They were provided with an explanation about the research purpose, how their confidentiality and the privacy of their personal information would be assured, and that they could leave the study at any time with no negative consequences. The participants were told that their answers would be kept confidential and would be used for research purposes only. They were also assured that their privacy would be maintained in scientific publications. Those who agreed to participate in the study signed an informed consent form. The participants completed the questionnaire and the open-ended question as they understood it and at will, such that not everyone answered this question.

### 2.4. Data Analysis

The data received from the participants’ replies to the open-ended question were analyzed with a content analysis. In order to identify the meaningful and main themes in the text, the analysis process included several major stages, according to the interpretive interactionism approach [20,21,22,23]. In the first stage, the participants’ replies to the open-ended question were read several times by two of the researchers. All the texts were read successively, repeatedly, in order to receive a full picture. In the second stage, each researcher read the text of the interviews once again and divided the text into meaning/content units. In the third stage, comparisons were conducted between the meaning units received, discussions were held on suitable titles and where to include each statement, and the units were focused and reduced in order to construct two main themes. These themes reflected the studied phenomenon in its context while examining the interrelations within the social context of being in-patients at an internal care department of a hospital, and they highlighted the meanings of the participants’ experiences [20].

## 3. Findings

The first theme deals with the humane attitude of respect and the right to privacy. A subtheme is treating the patient with respect by providing an explanation of the disease and of the treatment methods. The second theme deals with the significance of the beliefs, values, and traditional medicine that are passed between the generations. The findings will be presented below with quotes of the participants that will support and demonstrate the topics discussed. Notably, the quotes from the participants’ replies were chosen for the purpose of illustration in order to clarify the findings. Nevertheless, not all the participants’ statements are quoted in order to avoid repetition.

### 3.1. First Theme: Humane Attitude of Respect and the Right to Privacy

The issue of respect was extremely important for the participants, and many of them addressed it. On the one hand, some of the participants expressed satisfaction and even admiration at their physicians’ attitudes and emphasized that the positive attitude generated confidence, as some of the participants said: 

S.H., female, 90 years old: “[I] am amazed by the positive attitude of the physicians. They call me at home…”.

I.S., male, 71 years old: “It all begins with the head of the department…There is a good atmosphere and it is passed on to the entire staff. I don’t know what my condition is yet and what awaits me, but I feel confident”.

N.R., female, 75 years old also noted the cultural sensitivity of the staff, who treated her well as a religious woman: “During physicians’ visits only female physicians came in to examine me…”.

On the other hand, the topic of maintaining the right to privacy arose as a major issue among the participants. The patients claimed that their privacy was not maintained by the treating staff:

Y.K., male, 66 years old: “Not everything should be spoken about aloud. I don’t need my roommate to hear about all my illnesses and what I’ve been through. The right to privacy should be maintained”. 

N.S., female, 72 years old: “It is better to speak to the patient in a separate room–to tell him about his condition, the treatments, and what guidance is needed”.

A.C., male, 76 years old: “Young physicians should be taught…to maintain privacy”.

Another topic highlighted by the participants was the negative attitude of the physicians towards them—for example: 

L.Y., male, 73 years old: “The physicians let the patients feel that they have no time by forming almost no connection with the patients. [I] feel disrespect by the physicians, they don’t listen to the patients, don’t offer help”.

A.A., male, 80 years old: “There is no one to talk to. I constantly have to argue with everyone. No one listens to me…It is important for the patient that he is listened to and that his requests are listened to, because he is familiar with his body”.

R.M. female, 70 years old: “I have nothing, culture makes [no] difference, they should only be humane and know how to care for everyone. I have no expectations anymore, I only want to be treated fairly”.

L.R., female, 72 years old: “Young physicians should learn that you are not subhuman [even] if you are not a doctor. A doctor comes [but] doesn’t introduce himself. But the examination was thorough…”.

The topic of respect was associated with the subtheme that relates to the explanations the physicians gave the patients about their conditions.

#### Subtheme: Treating the Patient Respectfully When Providing an Explanation about His Illness and Treatment Methods

The participants attributed a great significance to the medical staff’s explanations of their conditions, and some of the participants felt that the physicians kept their distance by not trying to explain in comprehensible language—for example:

L.P., female, 73 years old: “Medical jargon distances the patient from the doctor”. 

B.B., male, 72 years old: “It is important for the patient that the treatment and examinations given will be accompanied by many explanations about the treatment/examination, that the treatment will be given with persuasion rather than forcefully”.

The unclear explanations were related to ageist attitudes towards the older participants, and they also complained about the distance they felt because of certain technologies. The need to bridge the intergenerational gap between young physicians and older adult patients was summarized by some of the participants: 

Y.Y., female, 77 years old: “[It is necessary] to respect the previous generation. A lot can be learned from them about modesty, love of human beings, and dignity”.

A.P., male, 75 years old: “It is important to maintain respect for people, even though we’re old. To speak clearly…”.

U.R., male, 90 years old: “It is important that the patient is heard. Don’t look at me like someone who is nearly 90 years old. I have lots of experience in the school of life”.

B.R., female, 78 years old: “I, myself don’t know what I have. Each time anew someone has to explain patiently. Today young people speak very rapidly, look only at the computer. They say something but I don’t understand. I’m embarrassed to ask for fear that they will think that I’m a primitive woman…”.

S.Z., female, 77 years old: “It is important to avoid making things complicated. Not to deal only with bureaucracy. We don’t understand when they say to me: ‘A fax was sent’…”.

T.O., male, 78 years old: “[I] feel uncertain although I am supposed to be discharged–I feel that the patients are not given enough information about their recovery process”.

The distance sensed by the participants is associated with a cultural gap due to language difficulties. For instance, one of the participants, H.H., male, 66 years old, who spoke Arabic, linked this to an insult to his dignity: “It is important to be respectful, to respect a person’s intelligence. Sometimes people look at you as though you understand nothing. And they don’t let you ask questions. I grew up in Israel…but my Arabic…”.

Russian speakers also stressed their lack of understanding. For example, Y.D., female, 74 years old, noted: “They [staff members] don’t explain anything because [I] speak only Russian, even if there are Russian speakers…they don’t admit that they can speak Russian…I feel very weak…”.

However, sometimes, the auxiliary staff try to help with translating, and sometimes, written information was given in another language. 

Y.Y., female, 74 years old: “[I] speak Russian, [I] usually don’t understand the information explained…They don’t provide written information in Russian…Some of the staff who do speak Russian, such as the ‘auxiliary staff’, try to translate and help”. 

Z.R., male, 68 years old: “A written explanation in Russian was provided before procedures such as giving a blood transfusion, and a consent form for treatment was also given in Russian–and that was very helpful and gave confidence…”. 

### 3.2. Second Theme: Beliefs, Values, and Traditional Medicine That Are Passed among the Generations

The descriptions of participants’ culture-related beliefs and values focused on religious faith and its manifestations, a healthy life routine, mutual help in the community, and traditional medicine.

With regards to religious faith, the participants noted: 

R.A., male, 80 years old: “For a patient’s recovery it is important that a Rabbi come to his sickbed; a Rabbi is called over from the synagogue to pray and to recite a blessing. After the blessing charity is given to the synagogue”.

O.Z., male, 77 years old: “It is important to observe the Sabbath and to observe the religious dietary laws”.

D.G., female, 76 years old: “[I] know to how appreciate the good. Every Sabbath night I wish all my children and grandchildren a good week. It is good for one’s mood and also for one’s health. A healthy soul in a healthy body. My entire approach to life is very optimistic”. 

Faith in a healthy life routine was evident regarding food, athletics, and occupation, as some participants said: 

Y.A., female, 69 years old: “[A] healthy breakfast, exercise, I believe that you must exercise”.

N.Z, male, 81 years old: “Slim equals healthy”. 

A.D., male, 80 years old: “Working is good for your health. I worked until I was 79 and it kept me in shape”.

Believing in the “evil eye” was also mentioned by the participants—for instance: 

K.V., male, 80 years old: “When someone would feel bad or have a sudden scare–people would think that he was jinxed and would hold a ’ceremony against the evil eye’–they would take a pot with hot water and lead and know who sent the evil eye by the shapes formed by the hot lead”.

Another value was mutual help within different ethnic groups:

D.R., female, 77 years old: “In the Georgian culture it is very customary to help each other out, so when one of the family is sick the entire family would come and help”.

Y.B., male, 72 years old: “When someone is sick in the [Bukharan] community money is collected for him, people go to the Rabbi to collect money or hold atonement for someone who is sick”.

In addition, the participants mentioned sharing—for example:

M.M., female, 81 years old: “[You] have to share. Health and culture mean talking. Not holding things in. That leads to many illnesses”.

However, with regards to Arab culture, one of the participants, Y.A., male, 66 years old, noted: “The Arab population has many prejudices. If someone has a fever, they immediately assume that he has cancer. Everybody knows everything very quickly. So things must be kept secret and I’m in this hospital and not close to home. Because many people know me. There is lots of concealment in Arab society”.

Some of the participants referred to the knowledge of medications familiar to them from their culture. For instance: 

V.H., male, 71 years old: “The family would consult with grandma in matters of health and she always knew what should be taken for an illness…The new does not replace the old–it develops from it”.

R.A., female, 82 years old: “With regard to health, we have all kinds of plants that you drink for stomachache, throat pain…the children laugh about it but when the time comes they will do it too”.

In conclusion, the participants expressed their desires for an attitude of respect, especially in light of their older age, and that the medical staff caring for them would understand their culture. They also emphasized their need to receive an explanation about their illness and treatment methods.

## 4. Discussion

The topic of cultural sensitivity to the patient population in general and to hospital patients in particular is an important part of the relationship between the medical staff and the patients, and it affects both the processes of a medical diagnosis and patient recovery. Hospitalization leads to encounters between the medical staff and people with diverse cultural backgrounds, characterized by different beliefs, attitudes, and life philosophies [2,3,10,24].

The current study explored the perspective of older adult patients in internal care departments in Israeli hospitals who come from multiple ethnic groups and cultures, with regards to the association between culture and health, with the aim of hearing patients’ attitudes and views. This trend of patient-centered care has become very common in recent years and is based on respect for the patients and their expectations of care, corresponding to the patients’ needs and social–cultural world [25].

Indeed, one of the main themes uncovered in the study relates to patients’ need for a humane attitude of respect, and judging by their many statements on the topic, they appear to ascribe great significance to this. It is evident from the participants’ statements that they have conflicting views. On the one hand, many participants voiced satisfaction with the attitudes of the medical staff, which included access to the staff and understanding their religious and cultural needs, granting them confidence, and even facilitating their recovery [13,26]. Then again, many patients noted the distance they felt from the medical staff. Some of the patients did not understand Hebrew, as they spoke only Arabic, Russian, or Spanish. Indeed, one of the reasons for the gap formed between the patients and the medical staff was language and communication barriers that resulted in a lack of understanding of physicians’ explanations regarding patients’ illnesses, and some patients also did not understand the instructions regarding their care [5,14,27]. The lack of understanding generated patient concerns about discharging from the hospital, which led to low satisfaction with the quality of care, as well as a lack of compliance with the instructions [5,27].

In fact, the contents of the Patient’s Rights Act, 1996 [28] were not implemented. For instance, article 13 of the law defines that medical information that must be provided to patients both in order for them to provide informed consent and as a manifestation of the patient’s involvement in decision-making regarding their health and preferences while providing care that respects the patient. The statements of the participants in our study regarding communication difficulties with the physicians showed that they did not receive appropriate care as regards the support and quality of service. Moreover, the research participants also said that their privacy was affected by explicitly noting that young physicians should be taught to maintain patients’ privacy, refrain from speaking out loud, and even suggesting that conversations with patients should be held in a separate room to maintain their privacy. Hence, it seems that the contents of article 10 in the Patient’s Rights Act [28] regarding maintaining the patient’s respect and privacy in all stages of the treatment are not implemented, judging by the participants.

In addition, the research participants expressed their feelings that they were not receiving suitable care due to their cultural and social status, education, and occupation, which differed from those of the medical staff. For instance, a person who worked in sanitation did not understand the medical terminology or a person who was in special education as a child. Although both speak Hebrew, they did not understand the medical terms, which were unfamiliar to them and, in some cases, were even embarrassed to ask in order to receive a suitable explanation. They felt that the physicians did not pay them enough attention, because they were old. We did not see any differences between non-Jews, ultra-Orthodox, and Arabs in terms of their feelings about the physicians’ attitudes towards them. All claimed that the physicians spoke rapidly (even for Hebrew speakers) and used technologies that were unfamiliar to them, such as computers and faxes. They felt that they had no one to speak to, and some even said that they had no expectations regarding the attitudes towards them so long as the medical examination was professional. The participants specifically noted that these attitudes by the staff towards them were because they were old. Hence, it seems that the participants ascribed to physicians’ ageist attitudes. 

Ageism is associated with stereotypical thinking, stigma, and prejudice towards an individual or group in the context of their increasing age, and it is explained as a complex term with several meanings that include three components: a cognitive component consisting of beliefs and attributions concerning old age and older adults, an emotional component manifested in attitudes in favor of or against old age and aging, and a behavioral component manifested in practical attitudes towards older adults [29].

The older population requires various health services at a high frequency, and healthcare providers such as physicians and nurses see them often in their professional practice. Frequent contact with and care of older adults, who are particularly vulnerable due to chronic morbidity and various disabilities, might generate ageist attitudes among these workers. Indeed, studies conducted on ageism among physicians and nurses at hospitals and long-term care facilities show that most staff prefer to avoid caring for older adults due to negative attitudes and stereotypes towards them and a lack of knowledge about old age [30,31,32,33,34,35].

Moreover, ageism affects the medical staff in healthcare services with regards to the essence and quality of care provided. Both physicians and nurses are providing medical services to a growing number of older people due to the increased life expectancy, but the negative attitude of the medical staff appears to affect the quality of care. This is resulting in a decline in health services [36,37,38].

Similar to our research findings, whereby the participants ascribed to the manifestations of ageism displayed towards them mainly from young physicians, who at times did not even introduce themselves to older patients, as one participant said: “Don’t look at me like someone who is nearly 90 years old. I have lots of experience in the school of life”. This was also evident from a study conducted among medical interns [39] who expressed negative views towards older adults and was indicated by the older adults’ feelings [40]. In any case, although physicians often have more knowledge of aging processes than the rest of the medical staff, they still display ageist attitudes [40,41]. According to researchers [38,41], further education focusing on intergenerational contact by geriatricians and medical staff is needed in order to reduce ageist attitudes and could improve the situation. One of our participants stated explicitly: “Young physicians should be taught…”. The intergenerational gap should be bridged, as one participant said in a message to the physicians that it is necessary to honor the previous generation, since a lot can be learned from them about modesty, love of human beings, and respect.

Additional themes found in the current study are beliefs and values that pass between the generations, such as religious faith, healthy life routines based on different beliefs such as healthy food, maintaining one’s weight, exercising, and working, as well as mutual help in the community for the sick. Other types of traditional medicines known in the family from previous generations and capable of helping in certain situations were also noted. There is no doubt that, when evaluating the patient and his needs in a patient-centered approach, it is very important to be familiar with the person’s sociocultural background [25]. For instance, studies conducted in Ghana [42,43] showed that cultural belief systems significantly affect people’s health-seeking behaviors. Personal health beliefs affect the use of traditional medicine and have a major role in shaping the use of medical treatments. The researchers’ conclusion was that health-seeking behaviors must take into account one’s sociocultural inclinations and include an understanding of people’s cultural values and beliefs in order to provide health services that will be acceptable. Similarly, a study conducted in Singapore showed that older Chinese Singaporean women navigated between traditional and modern medical practices, biomedicine, and traditional Chinese medicine in their practices of maintaining well-being [44]. 

Hence, it is necessary to embrace a culturally appropriate approach while understanding the role of the traditional structure and of cultural beliefs. These conclusions are indeed compatible with Kleinman’s [45] model, which not only describes patients’ symptoms but also asks what affects their illness and their coping with the illness. The model relates to patients’ beliefs and values, their folklore, family relations, and education, which affect the treatment experience and coping with illness. The cultural background is indeed significant. Thus, a study examining the associations between the network structure, interaction, support, and social engagement and self-rated health among Jewish Israelis, Arab Israelis, and new immigrants from the former Soviet Union found that social networks impact health differently in different cultural groups and under conditions of social changes, such as migration later on in life [46]. Similarly, the results of a study conducted in four European countries (Hungary, Italy, Portugal, and Spain) showed that age and culture affect the perceived health state of older adults [47]. Indeed, the findings of the present study highlighted the relationship between age and culture among older people. Each cultural group has its own unique beliefs—for example, the ultra-Orthodox emphasize the consideration of the hospital staff for their religious needs. Additionally, people from ethnic groups spoke about health-related beliefs in their culture and expected the staff to recognize and appreciate them. However, all the participants in the present study had in common feelings about the ageist attitude of the staff towards them because of their older age.

### 4.1. Limitations

This study addressed the replies to an open-ended question at one point in time. Some of the patients’ replies were given before the workshops were held and some after the workshops by other patients, such that, in this type of study, it is not possible to conduct a comparison regarding the efficacy of the workshops. Notably, the patients’ replies were also not compared to other questionnaires completed by them in the same period, which might have further expanded the repertoire of answers.

### 4.2. Recommendations for Further Research 

In the context of Israel, during this period of the pandemic (COVID-19), there were two prominent and well-defined minority groups: the ultra-Orthodox Jewish community and the Arab population. The government was slow to recognize the unique position of these two groups regarding the pandemic. Public pressure eventually led to a response that was tailored to the ultra-Orthodox community, and during the month of Ramadan, a similar response was implemented among the Arab community [48,49]. Therefore, our recommendation is to conduct further research on the cultural aspects related to the pandemic in order to help understand these aspects and endeavors to eradicate the pandemic. It is also recommended to add a question asking with whom does the older person consult about health issues in his or her social environment.

## 5. Conclusions

The findings of the current study indicate the great importance attributed by the patients to their culture, as well as to the attention of the hospital staff to their needs. Practically, it is very significant to become familiar with the patients’ sociocultural world—that is, to get to know the culture of people from different ethnicities. Another conclusion was related to the perception of older adults: it is important to listen to older adults, to understand their needs and their cultural characteristics, in order to reduce ageist attitudes towards them, as well as to enhance trust between older patients and those from other ethnicities and physicians [15].

The contribution of this specific study is to recommend the training of professional staff to understand patients’ cultural backgrounds, beginning at the stage of diagnosis through treatment and to discharge from the hospital, with the purpose of improving their service. In direct consultation with the health staff caring for older adults, it is possible to promote health interventions for their good health and well-being.
